# Supplementary material for: Pooling saliva samples as an excellent option to increase the surveillance for SARS-CoV-2 when re-opening community settings
Source: PLoS One. 2022 Jan 25;17(1):e0263114. doi: 10.1371/journal.pone.0263114 (PMC8789121; doi:10.1371/journal.pone.0263114)
Supplement: Attachment — Submitted filename: Response to Reviewer Comments.pdf [file pone.0263114.s001.pdf]

Response to Reviewer Comments

Reviewer #1:

The authors covered the feasibility of pooling saliva samples for surveillance of SARS-CoV-2 in community settings. Overall, the manuscript is well-written, covers all the necessary details and data is presented appropriately. The manuscript can be accepted after addressing a couple of issues.

We acknowledge the supportive comments of the reviewer.

1. Method: RNA extraction and RT-qPCR
The authors said they have used QuickExtract DNA Extraction Solution, which is probably used to extract gDNA for direct PCR? Kindly clarify.

Although Quick Extract DNA Extraction Solution (QE) is a reagent employed to extract DNA, it has been shown that it is also suitable for RNA extraction, and the RNA obtained from this treatment can be directly used in RT-qPCR reactions (1). We, and others have reported the effectiveness of QE for diagnosis of SARS-CoV-2 using saliva or swabs as source of viral genome (2)(3).

References

1. Kouranova E, Forbes K, Zhao G, Warren J, Bartels A, Wu Y, et al. CRISPRs for Optimal Targeting: Delivery of CRISPR Components as DNA, RNA, and Protein into Cultured Cells and Single-Cell Embryos. Hum Gene Ther. 2016 Apr 19;27(6):464–75.
2. Moreno-Contreras J, Espinoza MA, Sandoval-Jaime C, Cantú-Cuevas MA, Barón-Olivares H, Ortiz-Orozco OD, et al. Saliva sampling and its direct lysis, an excellent option to increase the number of SARS-CoV-2 diagnostic tests in settings with supply shortages. J Clin Microbiol. 2020;58(10):1–6.
3. Ladha A, Joung J, Abudayyeh OO, Gootenberg JS, Zhang F. A 5-min RNA preparation method for COVID-19 detection with RT-qPCR. Medrxiv. 2020;1–3.

2. The text can benefit from a thorough proofreading since there are spelling, grammar and punctuation issues throughout the manuscript.

We are very sorry for the mistakes in the text, we have proofread the manuscript and corrected all the spelling, grammar, and punctuation.
